# Patterns and presentations of colorectal cancer at Komfo-Anokye teaching hospital Kumasi, Ghana

**DOI:** 10.11604/pamj.2017.28.121.12927

**Published:** 2017-10-06

**Authors:** Francis Agyemang-Yeboah, Joseph Yorke, Christian Obirikorang, Emmanuella Nsenbah Batu, Emmanuel Acheampong, Emmanuel Amankwaa Frempong, Enoch Odame Anto, Bright Amankwaa

**Affiliations:** 1 Department of Molecular Medicine, School of Medical Science, Kwame Nkrumah University of Science and Technology (KNUST), Kumasi, Ghana; 2 Department of Surgery, School of Medical Science Komfo Anokye Teaching Hospital, Kumasi, Ghana; 3 Department of Oncology, Komfo Anokye Teaching Hospital, Kumasi, Ghana

**Keywords:** Colorectal cancer, incidence, clinical patterns and presentations, rectal cancer Ghana

## Abstract

**Introduction:**

Colorectal cancer is a major cause of morbidity and mortality globally and its incidence is increasing in developing countries. This study determined the incidence, clinical features and the histopathological patterns of colorectal cancer at Komfo Anokye Teaching Hospital (KATH), Kumasi, Ghana.

**Methods:**

A retrospective review of all colorectal cancer cases over a six year period from (2009-2015) presented to the Surgical and Oncological Department of KATH. Patients' records were retrieved and information on their demographics, clinical and pathological presentations recorded.

**Results:**

In all, 221 cases of colorectal cancer were identified over the study period. The mean age was 54 ± 16.8 and ranged from 16 to 90 years. Sixteen (7.24%) had family history of cancer and the prevalence of comorbidities was (24.89%). The commonest clinical symptoms presented were weight loss (44.80%), bleeding per rectum (39.82%) and abdominal pain (38.91%) Majority of the patients presented with rectal cancers (48.87%). Microscopically, adenocarcinoma (68.33%) was the most common histopathological type. According to Tumour Node Metastasis (TNM) staging of cancer, majority of the patients 89(40.27%) were identified as being in late stage (TNM Stage III). The overall crude annual incidence was 4.62 per 100000 populations. The age specific standardized incidence rate was 7.93 per 100,000 population

**Conclusion:**

This study has clearly showed a high incidence in colorectal cancer at KATH, with similar trends in clinico-pathological patterns comparable to that of most African countries. These include predominance of rectal cancers, high incidence among younger people and delayed presentation of the disease at advanced stage.

## Introduction

Globally, colorectal cancer (CRC) is the third most common malignant neoplasm with an incidence of 1.23 million new cases per year and the fourth most common cause of cancer deaths [1]. In Africa, the disease is considered rare but this is no longer so [2]. Current publications indicate that the incidence of colorectal cancer is increasing in developing countries including Sub Sahara Africa especially in the urban centers [3, 4]. The rate seem to increase in numerous African countries which traditionally were recognized as low risk countries, including Nigeria [5], Ghana [6], Tunisia, [7] and Egypt [8]. In these areas, CRC now represents about 10-50% of all malignant tumours. Colorectal cancer in West Africa has a characteristic unique pattern with an early age of onset and mostly left-sided tumours [6, 9]. Adenocarcinoma accounts for more than 95% of colorectal cancers with a disproportionate distribution in different segments of the bowel [10]. In West Africa, about half of the cancers are reported in the rectum [9]. About 78% of rectal cancers have being observed to be within reach of the examining finger [11]. In Ghana, the number of new cases of colorectal cancer has increased by 8-fold per year from an average of 4.1 new cases in 1960s to an average of 32.6 new cases currently [6, 12]. Current studies show that colorectal cancer is no longer uncommon among the indigenous people of Ghana. Patients mostly present with late stage cancers that are mostly incurable therefore resulting in poor clinical outcome of treatment for colorectal cancer [6]. This can be attributed to lack of knowledge about the disease and ignorance of the importance of early reporting to the hospital for early diagnosis and treatment. There is also dearth of data on the current trends in colorectal cancer in Ghana. It was against this background that this study was carried out to explore the presentation patterns, the age and gender distribution, sites of colorectal cancers and the histological types of colorectal cancers seen over a 6 year period at the Komfo Anokye Teaching Hospital, Ghana so as to provide an updated knowledge on the current trends of colorectal cancer in Ghana.

## Methods

**Study design/setting**: A retrospective study where all colorectal cancer cases presented to the Surgical and Oncological Department of Komfo Anokye Teaching Hospital were reviewed. Komfo Anokye Teaching Hospital (KATH) is a tertiary referral teaching hospital located in Kumasi, the Regional capital of the Ashanti Region in Ghana with a total projected population of 4,780,380 (Ghana Statistical Service, 2010). It is the second largest Hospital in Ghana.

**Study population and selection**: The case files of all CRC patients managed at the Hospital from 2009 to 2015 were retrieved from the Medical records unit of the surgery department and the Oncology Department. A total of 221 cases were identified. The records were analysed for information on demographics, clinical and pathological variables including histological type, grade of tumour and staging based on the TNM. The type of treatment and follow-up were also analysed. Information on age at diagnosis, gender, tumour location, pathological type of tumour, treatment modality, family history of CRC, metastasis were also reviewed.

**Inclusion criteria**: Records of patients, with complete clinical examination, indicating the presence of malignant tumours in the large bowel were included.

**Exclusion criteria**: Patients with other large bowel conditions and histopathologically confirmed non-malignant tumours were essentially excluded.

**Statistical analysis**: Data entry and analysis were performed using IBM statistical package for social science (SPSS) version 20. Descriptive statistics were performed for demographics variables, expressed as mean and standard deviation in the case of continuous variable with normal distribution. Association of clinical presentation with tumour location and stage was done using univariate and multivariable logistic regression analysis.

## Results

**Figure 1:**
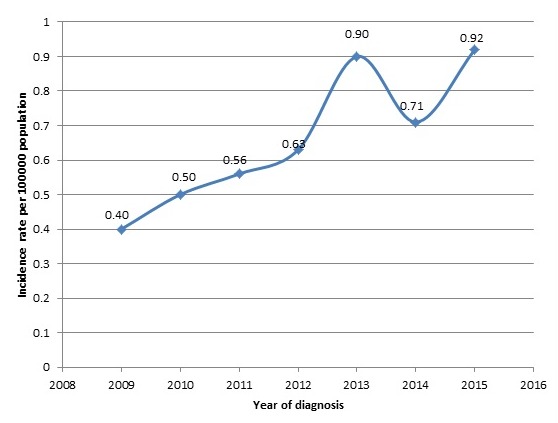
The incidence rate of colorectal cancer at KATH from 2009 to 2015

From this study, 221 cases of colorectal cancer were identified. The mean age of the study participants was 54 ± 16.8 which ranges from 16 to 90 years. Fifty (50) of the participants (22.6%) were less than 40 years and the majority (58) fell within the age group of 51-60 (26.2%). The majority of the studied participants 127(57.5%) were females (58%) and more than half 133(60%) were in the informal occupation sector. Most of them were married (63%) and Christians (85%). Lifestyle characteristics of the studied participants showed the prevalence of comorbidities at (24.89%). Eighteen 18 (8.14%), of the participants were diabetics, 44 were hypertensive (19.91%) and 11 had both DM/HPT (4.07%). A few of the participants 9 (4.07%) had other comorbidities such as asthma, HIV, hepatitis B, sickle cell disease and fibroid. Of the 221 cases, 16(7.24%) had family history of cancer, 21(9.50%) were alcoholics, 11(4.98%) were smokers and 9(4.07%) were both alcoholics and smokers Table 1. The duration of symptoms ranged from 3 days to 7 years and the median duration was 6 months (3-12 months). Majority of the subjects presented with 7-12 months of symptoms durations. Most of the subjects presented with more than one symptom. In all, most of the subjects presented with weight loss (44.80%), abdominal pain (38.90%) and bleeding per rectum (35.30%), which were the three most common presentations Table 2. Majority of the patients presented with rectal cancer cases 108 (48.87%), followed by colon 75(33.94%) and 7(3.17%) had tumors in more than one site. The rectum 96(43.40%) was the most frequent site for colorectal cancer, followed by the caecum 35 (15.80%) and sigmoid colon 22(10.00%). Microscopically, adenocarcinoma was the most common histopathological tumour in 151 (68.33%) patients and 104 (47.10%) of the tumors were moderately differentiated According to the TNM staging of cancer, 89(40.27%) were identified as being in late stage (TNM Stage III) and only 13 (5.98%) were in stage 1 Table 3. The overall crude incidence was 4.62 per 100000 populations per annum. Females had an incidence of 5.15 and that of males was 4.06 per 100000 populations. Within the age groupings, females within the age group of 60-69 and males within the age group70-79 had the highest incidence of (36.89) and (33.46) respectively. For both sexes, the highest incidence (34.42) was within the age groups of 60-69 and 70-79. The age specific standardized incidence rate using WHO world population as standard was 7.93 per 100,000 populations **Table 4**. Abdominal pain, constipation, anorexia, change in bowel habit, bleeding per rectum and anaemia had statistically significant associations (p < 0.05) in both univariate and multivariate analysis. With the exception of abdominal pain, all other parameters had reduced odds of association with colon cancers Table 5. Of all the symptoms, weight loss had the highest odds (1.775 and 2.077) of association with late stage tumours in both univariate and multivariate analysis and the association was statistically significant (p = 0.039) Table 6. The annual incidence rate of colorectal cancer increased steadily from 2009 to 2012 (0.4, 0.5, 0.56 and 0.63 per 100000 population). From 2012, there was a sharp increase incidence followed by a sharp decrease in incidence in 2014 to 0.71 per 100000 populations. Then in 2015 the incidence increased to 0.92 per 100000 populations. The overall crude annual incidence of colorectal cancer at KATH was 4.62 per 100000 populations Figure 1.

**Table 1:** Socio-demographic and lifestyle characteristics of study participants

Variable	Frequency n (%)	Variable	Frequency n (%)
**Age( Mean, SD)**	**54 ± 16.8**	**Presence of Comorbidities**	
≤40	50(22.60%)	Yes	55(24.89%)
41-50	31(14.00%)	No	166(75.11%)
51-60	58(26.20%)	**Family History**	
61-70	43(19.50%)	Yes	16(7.24%)
>70	39(17.60%)	No	205(92.76%)
**Gender**		**Diabetes**	
Male	94(42.50%)	Yes	18(8.14%)
Female	127(57.50%)	No	203(91.86%)
**Occupation**		**Hypertension**	
Formal	29(13.10%)	Yes	44(19.91%)
Informal	133(60.20%)	No	177(80.09%)
Student	11(5.00%)	**Both DM/HPT**	11(4.98%)
Retired	32(14.50%)	**Others**	9(4.07%)
Unemployed	16(7.20%)	**Alcoholic**	
**Marital Status**		Yes	21(9.50%)
Single	27(12.20%)	No	200(90.50%)
Married	140(63.30%)	**Smoker**	
Divorced	21(9.50%)	Yes	11(4.98%)
Widowed	33(14.90%)	No	210(95.02%)
**Religion**		**Both Alcool/Smoker**	9(4.07%)
Christian	187(84.60%)		
Muslim	32(14.50%)		
Traditional	2(0.90%)		

sd; standard deviation, others include asthma, HIV, hepatitis B, sickle cell disease and fibroid

**Table 2:** Clinical parameters of colorectal cancer among the study participant

Variable	Frequency n (%)	Variable	Frequencyn (%)
**Year of Diagnosis**		**Chemotherapy**	
2009	19(8.60%)	Yes	103(46.61%)
2010	24(10.90%)	No	118(53.39%)
2011	27(12.20%)	**Radiotherapy**	
2012	30(13.60%)	Yes	55(24.89%)
2013	43(19.50%)	No	166(75.11%)
2014	34(15.40%)	Both Chemo and Radio therapy	43(19.46%)
2015	44(19.90%)	Types of surgical operation Performed	
**Duration of Symptoms (months)**	Median 6(3-12)	Right Hemi colectomy	26(17.93%)
<6	95(42.99%)	Left Hemi colectomy	3(2.07%)
6 to 12	85(38.46%)	Sigmoid colectomy	14(9.66%)
>12	41(18.55%)	Transverse Colectomy	1(0.07%)
**Clinical presentations**		Low Anterior Resection ( LAR)	16(11.03%)
Abdominal Pain	86(38.91%)	Abdominal Peritoneal Resection (APR)	6(4.14%)
Constipation	70(31.67%)	Colostomy	63(43.45%)
Anorexia	45(20.36%)	Hartman's Procedure	13(8.97%)
Weight loss	99(44.80%)	Others	3(2.07%)
Change in bowel habit	46(20.81%)	**Anthropometric Measures**	
Bleeding per rectum	88(39.82%)	Body Mass Index(BMI) ( X^2^± SD)	**21.36± 5.77**
Hematochezia	64(28.96%)	Underweight	77(34.84%)
Anaemia	35(15.84%)	Normal	90(40.72%)
**Treatment Modalities**		Overweight	32(14.48%)
**Surgery**		Obese	22(9.95%)
Yes	145(65.61%)	**Patient Status**	
No	76(34.39%)	Alive	33(14.93%)
**Nature of operation**		Dead	103(46.61%)
Emergency	49(33.79%)	Lost to follow up	85(38.46%)
Elective	96(66.21%)		

**Table 3:** Pathological features of colorectal cancer at KATH

Variable	Frequency n (%)	Variable	Frequency n (%)
**Tumour Location**		**Histological Type of Cancers**	
Colon	75(33.94%)	Adenocarcinoma	151(68.33%)
Rectal	108(48.87%)	Mucinous Adenocarcinoma	26(11.76%)
Anorectal	18(8.14%)	Signet Ring Cell carcinoma	8(3.62%)
Anal	13(5.88%)	Squamous Cell Carcinoma	14(6.33%)
Multiple sites	7(3.17%)	Neuroendocrine Carcinoma	3(1.36%)
**Histological Grade**		Non Hodgkin Lymphoma	1(0.45%)
Well differentiated	51(23.1%)	Rbdomyosarcoma	1(0.45%)
Moderately differentiated	104(47.10%)	Others	4(1.81%)
Poorly differentiated	25(11.30%)	Not Stated/Unknown	13(5.88%)
Undifferentiated	24(10.90%)	**Anatomical Site of Tumour**	
Unknown	17(7.70%)	Caecum	35(15.80%)
**Tumour Stage**		Ascending Colon	10(4.50%)
stage I	13(5.98%)	Transverse Colon	1(0.50%)
stage II	64(28.96%)	Descending Colon	3(1.40%)
Stage III	89(40.27%)	Sigmoid Colon	22(10.00%)
Stage IV	36(16.29%)	Rectosigmoid	12(5.40%)
Blank	19(8.59%)	Rectum	96(43.40%)
		Anorectum	18(8.10%)
		Anus	13(5.90%)
		More than 1 site	11(5.00%)

**Table 4:** Age specific incidence and age standardised incidence rate of colorectal cancer at KATH

Variables	Age specific crude incidence rate per			Age standardised
	100, 000 population			incidence rate
Age Grouping(years)	Male	Female	Both Sexes	Both sexes
0-9	0	0	0	0.00
10-19	0.55	0.91	0.73	0.12
20-29	1.7	0.84	1.24	0.20
30-39	4.08	4.6	4.36	0.64
40-49	5.52	7.09	6.33	0.80
50-59	18.81	26.65	22.92	2.27
60-69	31.65	36.89	34.42	2.30
70-79	33.46	35.09	34.4	1.28
80-89	26.42	19.66	22.18	0.30
90-99	0	3.93	7.35	0.01
Overall	4.06	5.15	4.62	7.93

**Table 5:** Association of clinical symptoms with tumour location

	Univariate analysis			Multiple		
Symptoms	OR	95% CI	P Value	OR	95% CI	P Value
Abdominal Pain	4.61	(2.54 - 8.35)	0.0001	8.58	(3.701 - 19.876)	0.0001
constipation	0.51	(0.27 - 0.97)	0.041	0.35	(0.149 - 0.803)	0.013
Anorexia	0.35	(0.155 - 0.801)	0.013	0.26	(0.086 - 0.784)	0.017
Weight loss	0.58	(0.326 - 1.025)	0.061	0.85	(0.359 - 2.007)	0.71
Change in bowel habit	0.34	(0.149 - 0 .771)	0.01	0.35	(0.133 - 0.901)	0.03
Bleeding per rectum	0.13	(0.064-0.281)	0.0001	0.16	(0.071- 0.381)	0.0001
Haematochezia	0.55	(0.289- 1.062)	0.075	0.83	(0.376 -1.853)	0.656
Anaemia	0.35	(0.139- 0.887)	0.027	0.24	(0.071 - 0.790)	0.019

**Table 6:** Association of clinical symptoms with late stage tumour

	Univariate Analysis			Multiple		
Symptoms	OR	95% CI	P Value	OR	95% CI	P Value
Abdominal Pain	0.978	(0.548-1.744)	0.939	0.831	(0.44-1.57)	0.569
constipation	0.961	(0.5371.805)	0.985	0.897	(0.469-1.716)	0.743
Anorexia	1.104	(0.544-2.241)	0.784	0.755	(0.33-1.729)	0.507
Weight loss	1.775	(1.001-3.150)	0.05	2.077	(1.036-4.164)	0.039
Change in bowel habit	0.838	(0.415-1.694)	0.623	0.75	(0.361-1.562)	0.443
Bleeding per rectum	1.141	(0.639-2.036)	0.656	0.943	(0.504-1.763)	0.853
Haematochezia	1.287	( 0.6932.389)	0.424	1.274	(0.657-2.471)	0.473
Anaemia	1.438	( 0.6773.054)	0.344	1.204	(0.536-2.702)	0.653

## Discussion

The clinicopathological patterns of colorectal cancer have been reported to vary in different geographical regions. In the Western world, colorectal cancer occurs mostly in the elderly with most being diagnosed after the age of 50 years [13] whereas the disease in African population tends to present at a young age with advanced aggressive disease with poor prognosis [14]. In this retrospective study, we review the clinicopathological patterns and presentations of colorectal cancer at Komfo Anokye Teaching Hospital. The mean age of participants (54 ± 16.8) reported in this study is similar to findings in other African studies which reported mean ages of 53 year in Nigeria and 50.8 years in Burundi respectively [15, 16]. The observed mean age in this study appears younger than the age described in most developed countries [17, 18]. In the United States, the median age at diagnosis was 70 years. The peak age of presentation in this study was in the age range 51-60 years [18]. The probability of being diagnosed with colorectal cancer increases after 40 years of age [19]. In Africa, colorectal cancer mostly affects the young (< 40 years). Colorectal cancer incidence among the young (age less than 40 years) was 22% in this study, which is similar to findings in Egypt by Gado et al (2014) [20] where 25% of CRC occurred in patients aged less than 40 years. Higher incidence have been reported by Abdalla et al (2007) [21] in Khartoum hospital where 35.4% of patients were 40 years or less. Whereas in the Western countries, the incidence is about 2-6% [22]. These observations strongly indicates that colorectal cancer in Middle East and Africa seem to be more common in the young than in Western countries. Colorectal cancer in the younger age group has been shown to present with diagnostic and therapeutic problems and the prognosis tends to be less favorable [14].

The increasing incidence of CRC in the young in low-risk communities necessitates family screening for genetic mutations since genetic factors may play an important role in the development of this disease. This makes early detection and management an important measure in order to reduce incidence and mortality. On the other hand, it may be related to improvements in medical interventions and or to dietary factors since the young Africans tend to live more westernized life-style. Hence policy makers, in the implimentation of primary and secondary preventive measures should include the screening of young people who maybe at risk of developing colorectal cancer in Africa. Contrary to prevoius studies in Africa [6, 23], the gender prevalence of CRC in our study was in favour of females, with male: female ratio of 1:1.3 as compared to other studies which reported higher prevalence in males. Globally, incidence rates are considerably higher in males than in females [24]. This has beeen attributed to the higher adoption of certain risk behaviors associated with colorectal cancer such as: smoking, heavy alcohol consumption and obesity in men [13, 25]. The decreased incidence of colorectal cancer in women and female animals has been linked to a role of female hormones. In vitro evidence has demonstrated that estrogen regulates the cell growth of colonic mucosa and inhibits cell proliferation of colonic tumors through binding to estrogen receptors [26]. The current findings of higher prevalence of CRC in females could be due to the high prevalence of obesity among females in Ghana or high partronage of hospital attendance by females compared to males in our local setting since females are more conscoius of their health and hence report to hospital for the least discomfort they expericence. The prevalence of comorbidities among colorectal cancer patients in this current study was 24.9% (Table 1). A retrospective study by Van Leersum et al (2013) in south of Netherlands recorded an increase prevalence rate from 47% to 62% [27]. Other findings from Van Leersum studies indicated that hypertension and cardiovascular diseases were most prevalent comorbidities and this supports results from our current study where hypertension (19.9%) was the most prevalent comorbidity among the CRC patients. Risk of developing colorectal cancer is high for patients with a positive family history or underlying predisposing condition like ulcerative colitis. In this study, a family history of colorectal cancer was reported in 16 (7.2%) of cases, a figure which is higher than 5.4% and 4.3% reported by Chalya et al (2013) [28] in Tunisia and Azadeh et al (2007) [29] in Iran but similar to findings by kumar et al (2015) [30] in Oman who recorded 8.6%. This suggests that genetic factors may be playing an important role in the development of this disease in our country.

Majority of the patients presented with symptoms of weight loss 44.8%, bleeding per rectum 39.8% and abdominal pain 38.9% (Table 2). In a study conducted in Accra, Ghana, bleeding per rectum was the commonest symptom [6] which concurs with studies in other developing countries [31]. According to Giordano and Jatoi [32] the prevalence of weight loss is higher in patients with late stage cancers. Tisadale (2009) explained that tumor and host factors are responsible for the progressive attropy of adipose tissue and skeletal muscle that results in weight loss in about 50% of cancer patients [33]. The overall mortality rate in the present study was 46.6%, a figure which is higher than 6.1% reported by Dakubo et al (2010) in Ghana [6] and 10.% reported by Chyla et al (2013) [28]. The high mortality rate in our study may be attributed to the fact that most patients present with late staged cancers. Findings from this study also shows that the major anatomical site of CRC was the rectum (43.4%), followed by the caecum (15.8%) and sigmoid colon (10%) (Table 3). This trend is supported by findings from a systematic review study where authors reported that the major anatomical site of CRC in sub saharan Africa was the rectum (in 46% of cases), followed by the caecum (17%) [34]. This trend is also similar to findings in a retrospective study by Abdalla et al (2007) in Sudan [21] but contrasts with the right-side preponderance (proximal shift) reported by Guraya and Eltinay, (2006) [35] in Saudi Arabia and in developed countries [36]. Majority of the patients presented with late stage cancers (Stage III, 40.3%). Adenocarcinoma was the most common histological type (68.3%) with moderately differenciatiated tumours accounting for (47.8%) of the cases. This finding is in agreement with studies by Missaoui et al (2010) and Chalya et al (2013) [25, 28] who reported similar histopathological patterns. Many reasons have been ascribed to the late presentation of patients with CRC. In our local settings, the reason could be that warning symptoms may be taken for granted by many patients who may not relate them to serious disease and so be ignored. Other reasons could be ignorance with misconception on the cause of CRC, low standard of education, insufficient diagnostic and therapeutic equipment and lack of screening and awearness creating programs in this region.

There is a marked variation in the incidence of colorectal cancer worldwide, with Western countries having a high rate compared to Africa [13, 37]. However, a rising incidence of colorectal cancer has been reported from various parts of Africa which were considered low incidence areas [23, 38]. Findings from this study show that, the overall crude annual incidence of colorectal cancer at KATH was 4.62 per 100000 populations. The age specific standardized incidence rate using WHO world population as standard was 7.93 per 100,000 populations (Table 4). These findings are comparatively lower than results by Dakubo et al (2010) in Accra, Ghana where the number of new cases seen annually was 32.8 in a population of about 3,000,000 [6] The crude incidence rate was 11.18 per 100,000 population in both sexes combined. According to a study by Laryea et al (2014) on cancer incidence in Ghana, using the population bassed cancer registry at KATH, the estimates for crude incidence and age standardized incidence of colorectal cancer was in the range of 0.1 to 0.3 per 100,000 [39], Compared to our current estimates, there have been a drastic increase in the incidence of colorectal cancer over the years in Kumasi, although not as high as reported in the developed countries. Incidence rates reported in other developing countries are similar to our current findings, the age standardized incidence rate for colorectal cancer in United Arab Emirates was 6.8/100,000 person per year, which is similar to our current estimate. Previous studies have shown incidence reports of 1.7 cases per 100,000 person per year in some Western African countries to 51.7 cases per 100,000 person per year in North America [40]. In the current study, we have shown that there are significant differences in the clinical presentations of patients depending on tumour location and tumor staging. Finding from this study (Table 5) shows that, abdominal pain is significantly associated with colon cancers whereas constipation, anorexia, change in bowel habit, bleeding per rectum and anaemia are more prevalent in non colon cancers (rectum, anorectum and anal cancers) in both univariate and multivariate analysis. This agress with results from Peedikayi et al (2009) [41] in India who reported bleeding per rectum and constipation to be associted with distal CRC and abdominal pain with proximal CRC. Studies by Vanek et al (1986) and Alexiusdottir et al (2012) reported that, colon cancers were associated with higher incidence of anaemia [42, 43]. A study by Ben-Isah et al (2013) indicated that patients in the late TNM stages presented with significantly more weight loss (p = 0.040) [44]. This is consistent with the observation in this current study which showed that, weight loss is significantly associated with late TNM stage cancers (Table 6). Other studies have also reported change in bowel habit and abdominal pain to be associated with late TNM stage tumours and bleeding per rectum with early TNM stage [43, 45], however this trend was not observed in this current study.

## Conclusion

Our study has established that, there is a progressive but steady increase in the incidence of colorectal cancer at KATH, Ghana, with similar trends in clinico-pathological patterns and presentations as that of most African countries. These patterns include predominance of rectal cancers, high incidence in the younger people and delayed presentation of the disease in an advanced stage. Peculiar to our setting was the predominance of females, majority with symptom of weight loss and moderately differentiated adenocarcinomas. The increasing incidence in the young necessitates family screening for genetic mutations since genetic factors may play an important role in the development of this disease. A nationwide health education to create awareness among communities to increase care seeking behavior is also highly recommended.

### What is known about this topic

- Colorectal cancer is mostly known as a disease of developed countries with a Western culture;
- In Africa, colorectal cancer is considered rare but this is no longer so. Incidence of this disease is increasing in developing countries including Sub Sahara Africa especially in the urban centers;
- Colorectal cancer in West Africa has a characteristic unique pattern with an early age of onset and mostly left-sided tumours. Common symptoms include rectal bleeding, abdominal pain, altered bowel habits and involuntary weight loss.

### What this study adds

- This study reports a progressive increase in the incidence of colorectal cancer at KATH, in Ghana which supports the literature that incidence of this cancer is on the rise in Africa and other developing countries;
- The commonest clinical symptoms presented were weight loss, bleeding per rectum and abdominal pain due to late presentation hence resulting in high mortality rate in our study population;
- There was a high incidence of colorectal cancer among females and younger people below the age of 40 which may implicate genetic factors in the etiology in our population.

## Competing interests

The authors declare no competing interests.
